# Cognitive Change and Relaxation as Key Mechanisms of Treatment Outcome in Chronic Pain: Evidence From Routine Care

**DOI:** 10.3389/fpsyt.2021.617871

**Published:** 2021-08-03

**Authors:** Matthias Feldmann, Hauke Jeldrik Hein, Ulrich Voderholzer, Robert Doerr, Thomas Hoff, Gernot Langs, Philipp Herzog, Tim Kaiser, Winfried Rief, Jenny Riecke, Eva-Lotta Brakemeier

**Affiliations:** ^1^Department of Clinical Psychology and Psychotherapy, Philipps-University Marburg, Marburg, Germany; ^2^Department of Clinical Psychology and Psychotherapy, University of Greifswald, Greifswald, Germany; ^3^Schoen Clinic Roseneck, Prien am Chiemsee, Germany; ^4^Department of Psychiatry and Psychotherapy, University Hospital Munich, Munich, Germany; ^5^Schoen Clinic Berchtesgadener Land, Schönau am Königsee, Germany; ^6^Schoen Clinic Bad Bramstedt, Bad Bramstedt, Germany; ^7^Department of Psychiatry and Psychotherapy, University of Lübeck, Lübeck, Germany

**Keywords:** chronic pain, coping skills, mechanisms of change, naturalistic, multidisciplinary pain treatment, cognitive behavioral therapy, effectiveness

## Abstract

Despite effective treatment approaches within the cognitive behavioral framework general treatment effects for chronic pain are rather small to very small. Translation from efficacy trials to naturalistic settings is questionable. There is an urgent need to improve the effectiveness of well-established treatments, such as cognitive-behavior therapy (CBT) and the investigation of mechanisms of change is a promising opportunity. We performed secondary data analysis from routine data of 1,440 chronic pain patients. Patients received CBT in a multidisciplinary setting in two inpatient clinics. Effect sizes and reliable change indices were computed for pain-related disability and depression. The associations between changes in the use of different pain coping skills (cognitive restructuring, activity despite pain, relaxation techniques and mental distraction) and changes in clinical outcomes were analyzed in structural equation models. Pre–post effect sizes range from *g* = 0.47 (disability) to *g* = 0.89 (depression). Changes in the use of cognitive restructuring, relaxation and to a lesser degree mental distraction were associated with changes in disability and depression. Effects from randomized trials can be translated to naturalistic settings. The results complement experimental research on mechanisms of change in the treatment of chronic pain and indicate an important role of cognitive change and relaxation as mechanisms of change. Our findings cautiously suggest that clinicians should optimize these processes in chronic pain patients to reduce their physical and emotional disability.

## Introduction

For decades, the Global Burden of Disease Study has ranked chronic pain as the world's greatest cause of disability. Globally, it is the leading cause of years lived with disability [YLD, (1)]. Its negative impact on the quality of life is the highest of all diseases (2, 3) and it brings severe challenges for patients (4, 5): Patients diagnosed with chronic pain sleep worse (6), have an increased suicidal tendency (7), a deteriorated sexuality (8) and an increased probability of a comorbid depression (9), which also represents a major health problem (10). The management of chronic pain is characterized by a range of different treatments e.g., medication, exercise or cognitive behavioral therapy [CBT, (11)]. To date, a biopsychosocial perspective is broadly accepted and is best reflected by a multidisciplinary treatment approach (12, 13). There is ample evidence that psychosocial factors such as pain-related fear are more important in explaining disability than pain itself (14). The cognitive-behavioral perspective explicitly targets these factors and includes a variety of well-established psychological treatment options for chronic pain (15).

The main goal of CBT in treating chronic pain is to increase the patient's functionality (16). Turk and Flor (17) categorize three CBT-specific treatment approaches for chronic pain—operant, cognitive, and respondent—each targeting different mechanisms of change. The operant approach focuses on operant learning mechanisms by reducing maladaptive pain behavior (e.g., avoiding activity due to fear of injury) and building up positive activities. In the cognitive approach, cognitive restructuring is performed to change evaluative aspects of pain (e.g., “I can't do anything about my pain”) and generate more positive pain-related expectations. The respondent approach intends to directly modify the response to pain-related stimuli—muscle tension and anxiety—by means of relaxation techniques (11). In CBT, patients are taught to target these three response systems by adaptive pain coping skills (18, 19). There is a large body of research focusing on different pain coping skills that target one of the three different response systems (17): Being active despite one's own pain is important and can improve pain symptoms via the operant response system (20, 21). Cognitive restructuring improves pain coping by targeting the cognitive response system (22, 23) and applying relaxation techniques targets the respondent system (24, 25). Evidence on mental distraction, another pain coping skill routinely used and originally included in the cognitive approach (17), is mixed: mental distraction may be considered helpful by patients (26) but could aggravate the experience of pain in the long run (27, 28). Coping self-affirmations may also not have any positive effect on pain coping (29). In summary, different coping skills seem to be heterogeneously effective in managing pain (29, 30).

Despite effective treatment approaches within the cognitive behavioral framework (31–33) general treatment effects are rather small to very small (34). Furthermore, long-term stability is questionable (35, 36) as well as the generalizability of efficacy trials on the effects on everyday clinical practice (37). There is an urgent need to improve the treatment for patients with chronic pain. Since an effective treatment of chronic pain remains a challenge, Morley et al. call for a paradigm shift in research (38). They promote complements to efficacy trials (e.g., randomized controlled trials, RCTs) and emphasize, among others, secondary data analysis. Accordingly, practice-based evidence (PBE) research methods can make an important contribution to treatment improvement (39). Although observational PBE studies do not allow to draw causal conclusions (40), they can identify treatment elements associated with better clinical outcomes for a wide range of patients (41). In addition, they usually incorporate a high sample size of patients, yield a high external validity and are cost-efficient (42). PBE should therefore have a firm place in research (39) and can also be successfully implemented in the field of chronic pain (43).

In order to enhance the effectiveness of CBT in the treatment of chronic pain, research on the driving mechanisms of change is vital (44). Routine clinical data can contribute to this (45–47). The effectiveness of coping skills has been assessed experimentally or in RCTs. While experiments usually have a high internal validity, they often only provide short-term results on acute pain. These results are difficult to transfer to clinically relevant outcomes like pain-related disability. Clinical RCTs do have the potential to test causal assumptions on clinically relevant outcomes. However, usually treatment arms consist of a bundle of interventions (48) and thus questions about the impact of specific pain coping skills remain unanswered. A direct comparison of the impact of different pain coping skills on clinical outcomes such as pain-related disability could therefore provide valuable information for possible treatment improvements. Assessing the impact of changes in mechanisms on changes in outcomes is a common technique to draw conclusions about their importance (44) and it is vital to compare multiple potential mechanisms of change in the same study (48). As different pain coping skills are thought to target different mechanisms of change, the investigation of associations between changes in the use of coping skills and changes in outcomes could help to answer the important question, which mechanisms of change are most essential in routine clinical care thereby increasing the effectiveness of CBT in the long run.

Analyzing a large routinely collected pre-post-data set from two large clinics specialized in chronic pain implementing a multidisciplinary pain treatment program, the first aim of this study is to investigate the effectiveness of routine clinical care in reducing pain-related disability and depression in chronic pain patients. The second goal of this study is to examine the associations between changes in the use of different pain coping skills and changes in clinical outcomes (pain-related disability and depression). We believe that a focus on the change in the use of pain coping skills can provide valuable insights into mechanisms of change involved in the treatment of chronic pain. The coping skills selected are based on the three approaches of Turk and Flor (17). We investigate the change of activity despite pain (operant approach); cognitive restructuring and mental distraction (cognitive approach); and relaxation (respondent approach). Based on the previous literature we expect that an improved use of cognitive restructuring, relaxation and activity despite pain are equally related to changes in clinical outcome measures. Furthermore, we hypothesize that an improvement in the use of mental distraction is to a lesser extent positively related to our outcomes.

## Method

### Procedure

Routine pre-post data from two large psychotherapeutic clinics in Germany were analyzed. Data was collected between 2013 and 2017. Patients completed self-report questionnaires immediately before and after treatment. Pain coping skill use and depression was measured in both clinics, while pain related disability was only routinely measured in clinic A.

### Patient Sample

Patients were included who had been treated for at least 4 weeks for chronic pain, who were diagnosed with F45.41 according to the ICD-10-GM (49) by trained clinical practitioners and who filled out the Questionnaire for the assessment of pain processing [“Fragebogen zur Erfassung der Schmerzverarbeitung,” FESV, (50)] at the beginning of the treatment. This led to a total sample size of *N* = 1,440, with *n* = 754 patients treated in clinic A and *n* = 686 in clinic B who were included in the analyses. All patients gave informed consent for their anonymous data to be used for research purposes. There were *n* = 661 (87.7%) complete cases for the analysis of associations between changes in pain coping skills and pain related disability in clinic A and none in clinic B, because pain related disability was only assessed in clinic A. There were *n* = 668 (88.6%) complete cases for the analysis of associations between changes in pain coping skills and depression in clinic A and *n* = 570 (83.1%) in clinic B. Full missing data specifications are included in Table 1.

**Table 1:** Missing data in both clinics.

	**FESV T1 *n* (%)**	**FESV T2 *n* (%)**	**PDI T1 *n* (%)**	**PDI T2 *n* (%)**	**PHQ T1 *n* (%)**	**PHQ T2 *n* (%)**
Clinic A (*N =* 754)	0 (0.0)	54 (7.2)	5 (0.7)	55 (7.3)	7 (0.9)	58 (7.7)
Clinic B (*N =* 686)	0 (0.0)	115 (16.8)	686 (100)	686 (100)	0 (0.0)	97 (14.1)

*PHQ-9, Patient Health Questionnaire; PDI, Pain Disability Index; FESV, Pain Management Questionnaire*.

### Multidisciplinary Treatment Program

Patients received a multidisciplinary treatment in one of two clinics specialized for chronic pain. Both clinics offered acute care and rehabilitative routine care. In Germany, rehabilitative care follows the goals of tertiary prevention and acute care of secondary prevention. The treatment was based on CBT for chronic pain intended to last for 5 weeks (rehabilitative care, *n* = 556) or 6 weeks (acute care, *n* = 884). In some cases, the duration of treatment has been extended in agreement with the healthcare provider. Patients received treatment for 28–84 days with a median of 41 days (*SD* = 8.1). Twice a week (on average 200 min per week) all patients participated in manualized CBT group therapy for chronic pain, in which they received pain-related psychoeducation and training for pain coping skills. Patients also received individual non-manualized CBT for at least 50 min per week. Patients received additional non-manualized CBT, depending on the ward in either a group setting or an individual setting. All CBT treatments were administered by trained therapists. There were medical visits at least once a week by a medical doctor and the head of the ward, group visits multiple times per week and daily contact with co-therapists. An individual treatment plan was tailored to each patient by a multidisciplinary team. Optional treatment components consisted of general group therapy; social competence training; group therapy for psychological comorbidities; physiotherapy; sport and movement therapy; mindfulness and relaxation group training; biofeedback; social counseling; arts and craft therapy; and body therapy. While psychological interventions were the main treatment focus, medication was administered according to the current national and international guidelines.

### Measurements

#### Pain Coping Skills

The FESV (50) is a well-established self-rating questionnaire that measures pain coping skills. It was specifically designed to assess the coping repertoire for chronic pain. The FESV has been found suitable for multipoint surveys (51). The FESV is based on empirical research about pain processing. It consists of three scales (cognitive pain coping, behavioral pain coping, and pain-related psychological impairment). Each scale has three subscales. For the present study, we used the cognitive pain coping scale and behavioral pain coping scale. Of their six subscales two were excluded because they are conceptually too close to the evaluated outcomes (*coping self-efficac*y, “When I feel pain, I am sure that I can deal with it” and *action planning*, “When I feel pain, I have a number of possibilities to fight it”). The remaining four scales measure the usage of active coping skills during the occurrence of pain: *cognitive restructuring* (e.g., “When I feel pain, I weigh it against the good sides of life,” Cronbach's α = 0.75 in the current study), *mental distraction* (e.g., “When I feel pain, I distract myself by listening to pleasant music,” α = 0.74), *activity despite pain* (e.g., “When I feel pain, I conceal them by just continuing with my work,” α = 0.77) and *relaxation techniques* (e.g., “When I feel pain, I apply a relaxation technique (e.g., autogenic training, progressive muscle relaxation),” α = 0.79). All items are formulated as statements to be answered on a six-point response scale ranging from 1 (not agree) to 6 (completely agree).We tested the factor structure of the subscales in the current dataset. Parallel analysis resulted in four components. All items but one loaded with at least 0.6 on their theoretically assumed factor. The item that did not sufficiently load on any factor originally belonged to the subscale *activity despite pain* (“When I feel pain, I actively seek contact with other people to distract myself.”) and was excluded from further analysis, which led to an increased Cronbach‘s α = 0.83. In order to avoid a logical tautology, we excluded one of the four items of the cognitive restructuring scale that implied a positive therapy outcome (“When I experience pain, I tell myself that I can cope with it much better than before”). This led to a reduced Cronbach's α = 0.67. Overall, Cronbach's α was slightly lower than in comparable studies where it ranges from Cronbach's α = 0.78 (*cognitive restructuring*) to Cronbach's α = 0.85 (*mental distraction and activity despite pain*) (52). The reliabilities of the difference scores as calculated in the sample using the formula in Gollwitzer et al. (53) were between α = 0.40 (*cognitive restructuring*) and α = 0.65 (*activity despite pain*).

#### Pain Related Disability

The Pain Disability Index [PDI, (54)] is a well-established seven-item self-rating questionnaire that assesses patients' current perceived level of disability in seven life domains (e.g., social activity; occupation; or self care) with one item each (55). The seven items are assessed on a 0 (no disability)–10 (worst disability) numeric rating scale, with the sum score ranging from 0 to 70. The internal consistency in this sample was Cronbach's α = 0.81 which is consistent with the results in comparable samples (56). The reliability of the difference score in the sample was α = 0.68.

#### Depression

Depression was measured with the German version (57) of the Patient Health Questionnaire-9 [PHQ-9, (58)], which has been successfully validated for the purpose of measuring treatment outcomes in depression (59). The self-rating questionaire consists of 9 items (e.g., “Feeling tired or having little energy.”) each with four possible answers ranging from 0 (not at all) to 3 (nearly every day). The sum score (0–27) indicates the level of depression with a higher score indicating greater severity of depression. The internal consistency in this sample was α = 0.85 which is comparable with other studies (60). The reliability of the difference score in the sample was α = 0.75.

### Statistical Analysis

All statistical analyses were performed in R (61) using the packages tidyverse, lavaan and psych (62–64). We conducted *t*-tests between the two clinics for all continuous variables and chi-squared tests between the two clinics for all nominal variables.

#### Effect Sizes and Clinical Significance

Pre–post effect sizes (Hedge's g) were computed for all outcomes and pain coping skill variables for completers using the package effectsize (65). Additionally, last observation carried forward effect sizes (LOCF) were computed for the same variables in order to provide a more conservative effect size estimation based on the assumption that individuals with missing data did not show any improvement. Clinical significance and reliable change indices (66) were computed using the R package JTRCI (67). The reliabilities were based on German validation studies [PHQ-9: α = 0.88; PDI: α = 0.88, (57, 68)]. A PHQ-9 value of *M* = 3.3 [*SD* = 3.7, (60)] for depression was used as a norm for the healthy population, resulting in a cutoff for recovery of c = 7.2 where individuals were equally likely to belong to a healthy population and the pre-treatment sample in this study (69). A PDI score of *M* = 9.0 [*SD* = 12.6, (70)] reported in a German sample with at least one physical complaint was used as a norm for the healthy population for disability (*c* = 24.9).

#### Modeling the Associations Between Changes

We used structural equation modeling (SEM) to model associations between changes from pre-treatment to post-treatment. We modeled a single indicator latent change score (53, 71) for each of the four pain coping skill use scales and the two outcomes PDI and PHQ-9 using their sum scores. Latent change scores seperate the part of the variance that remains constant between two points of measurement from the part that changes (71). Latent change scores offer advantages over manifest difference scores or residual change scores (53) and have been used in previous studies in which associations between changes over time were modeled (72, 73). Gollwitzer et al. (53) advocate the use of latent change scores with multiple indicators. However, we believe that the pain coping skill scales represent composites rather than single latent factors (74). In this case using a miss-specified measurement model with multiple indicators can lead to more biased estimates than using sum scores, even when measurement error is present (74) which is why we use sum scores as single indicators when modeling latent changes.

All latent change scores were included in the same model. To estimate the total associations between variables, we first created a model in which all correlations between pre-treatment variables and latent change variables were allowed. In a second step we checked whether the pain coping skills scores uniquely explained variance in the change scores of the outcomes. Therefore, in these models, each of the pain coping skill use change scores was regressed on each available outcome change score.

#### Comparison of the Models Between Clinics

A first SEM model was fitted using the data from clinic A. Here, both outcomes were available: PDI and PHQ-9. The data from clinic B was used for a partial replication: Model 2 was fitted using all data from clinic B and the PHQ-9 as the outcome.

In order to test for differences in the associations between changes between the two clinics, all data available was grouped by clinic. Here the PHQ-9 was used as the only outcome, as it was available in both clinics. Four models were fitted that varied in which parameters were fixed to be equal in both clinics: In Model A, the null model. all parameters could vary freely between the clinics. In Model B all regression coefficients were fixed to be the same between clinics. In Model C all regression coefficients and all covariances between latent variables were fixed to be the same between clinics and in Model D all parameters were fixed to be the same between clinics.

Model fit indices Akaike Information Criterion (AIC), Bayesian Information Criterion (BIC), Tucker-Lewis Index (TLI), Comparative Fit Index (CFI), Standardized Root Mean Residual (SRMR) and Root Squared Mean Error Average (RMSEA) were computed for all models. Additionally, nested model comparisons were performed.

#### Computation of SEM Models

All SEM models were fitted using lavaan (63) using full information maximum likelihood estimation to deal with missing data.

Sensitivity analyses were performed by using a robust maximum likelihood estimator with Huber-White standard errors and by using a different method for dealing with missing data: multiple imputation with the package MICE (75). Estimates from estimations on 100 imputed datasets were pooled using the package SEMTools (76).

## Results

### Sample Description

Patients were between 19 and 87 years old (*M* = 52.4; *SD* = 10.2). Of *N* = 1,440 patients, 1,070 (74.3%) were female. Patients were diagnosed with an average of 2.9 (*SD* = 1.2) mental disorders according to the ICD-10. A total of 333 patients (24.4%) were able to work before the treatment; 754 patients (52.4%) were treated in clinic A and 686 patients (47.6%) were treated in clinic B. Patients in clinic B differed from patients in clinic A in a number of variables: They were treated more often in rehabilitative care, were diagnosed with fewer mental disorders, were more often treated in an inpatient ward for the first time, were treated for a shorter period of time and had lower scores of depression at intake. An extended summary of patient characteristics in comparison between Clinics A and B are presented in Table 2.

**Table 2:** Sociodemographic and clinical characteristics as well as self-rating scores of the sample (*n* = 754 clinic A and *n* = 686 clinic B) at baseline.

**Characteristics**	**Clinic A (*n =* 754)**	**Clinic B (*n =* 686)**	***p^f^***
Age (years) *M* (SD)	52.1 (10.2)	52.6 (10.2)	<0.001
Sex *n* (%)			0.05
Male	177 (23.5)	193 (28.1)	
Female	577 (76.5)	493 (71.9)	
Educational score *M* (SD)^a^	2.8 (0.9)	2.4 (1.1)	<0.001
Number of mental Disorders *M* (SD)	3.1 (1.2)	2.7 (1.1)	<0.001
Frequent comorbidities *n* (%)^b^			
Depressive episode (F32)	160 (21.2)	122 (17.8)	0.12
Recurrent depressive disorder (F33)	570 (75.6)	487 (69.7)	0.01
Personality Disorder (F60)	104 (13.8)	63 (9.2)	0.008
Married *n* (%)	473 (63.4)	374 (60.7)	0.65
In a relationship *n* (%)	625 (83.8)	487 (79.1)	0.03
First inpatient treatment *n* (%)	399 (74.3)	499 (85.2)	<0.001
Occupational status *n* (%)			<0.001
Unemployed	104 (13.9)	115 (18.7)	
Retired	288 (38.6)	184 (29.9)	
Working at least part time	288 (38.6)	386 (46.5)	
Other	46 (6.2)	17 (2.8)	
Working ability *n* (%)	178 (23.8)	155 (25.2)	0.61
Outpatient psychotherapy *n* (%)	359 (48.1)	244 (39.6)	0.002
Outpatient psychiatric treatment *n* (%)	440 (59.0)	441 (71.6)	<0.001
Duration of treatment (in days)	44.9 (8.8)	39.7 (6.2)	<0.001
PHQ-9 score *M* (SD)^c^	15.4 (5.6)	13.9 (5.7)	<0.001
PDI score *M* (SD)^d^	40.6 (12.6)	-^g^	
FESV Cognitive Restructuring *M* (SD)^e^	3.2 (1.1)	3.3 (1.2)	0.03
FESV Mental Distraction *M* (SD)^e^	2.8 (1.1)	2.8 (1.2)	0.65
FESV Activity Despite Pain *M* (SD)^e^	2.8 (1.1)	3.0 (1, 2)	0.008
FESV Relaxation Techniques *M* (SD)^e^	2.9 (1.2)	3.1 (1.2)	<0.001

a *Based on the German school system; scale from 0 (no degree) to 4 (general qualification for university entrance);*

b *Diagnosis as given by practitioners according to ICD-10;*

c *PHQ-9, Patient Health Questionnaire, 9 items scale 0–3 per item;*

d *PDI, Pain Disability Index, seven items scale 0–10 per item, range 0–70;*

e *FESV, Pain Management Questionnaires, 1–6 per item;*

f *p-values derived from t-tests for continuous variables and chi-squared tests for categorical data for differences between clinics;*

g *this questionnaire was not routinely administered in clinic B*.

### Effectiveness

Across both clinics, effect sizes were large for depression [*g* = 0.89, 95% CI = [0.82, 0.95]] and medium for pain-related disability [*g* = 0.47, 95% CI = [0.39, 0.55]]. Using the more conservative LOCF estimates, effect sizes were still large for depression [LOCF *g* = 0.81, 95% CI = [0.75, 0.87]] and medium on pain-related disability [LOCF *g* = 0.45, 95% CI = [0.37, 0.52]]. Out of *n* = 754 patients available for the reliable change analysis in pain related disability, 65 (8.6%) recovered, 74 (9.8%) reliably improved, 83 (11.0%) recovered non-reliably, 446 (60.2%) remained unchanged, 28 (3.7%) deteriorated and 64 (8.5%) did not complete the post-treatment questionnaire. Examining reliable change in depression, 222 (15.4%) recovered, 256 (17.7%) improved reliably, 231 (16.0%) recovered non-reliably, 549 (38.1%) remained unchanged, 22 (1.5%) deteriorated and 160 (11.1%) did not complete the post-treatment assessment.

### SEM Models

#### Associations Between Changes in Pain Coping Skill Use

In clinic A all changes in pain coping skill use were positively correlated. Changes in the use of cognitive restructuring and relaxation were correlated with a large effect size (*r* = 0.49, 95% CI = [−0.43, 0.54]]. Changes in the use of mental distraction were correlated with medium effect sizes with changes in the use of cognitive restructuring [*r* = −0.32, 95% CI = [0.25, 0.38]] and relaxation [*r* = 0.36, 95% CI = [0.30, 0.43]]. There were small correlations between changes in the use of activity despite pain and the other pain coping skills (0.09 ≤ r ≤ 0.22). All bivariate correlations between baseline variables and change scores are presented in Appendix A.

#### Associations With Changes in Pain-Related Disability

In clinic A, higher reductions in pain-related disability were correlated with positive changes in the use of cognitive restructuring [r = −0.22, 95% CI = [−0.29, −0.15]], with positive changes in the use of relaxation technique [r = −0.24, 95% CI = [−0.31, −0.17]], and with positive changes in the use of mental distraction [r = −0.16, 95% CI = [−0.23; −0.09]]. All effect sizes were small to medium. Changes in activity despite pain were not correlated with changes in pain-related disability [r = −0.04, 95% CI = [−0.11, 0.03]].

**Figure 1:**
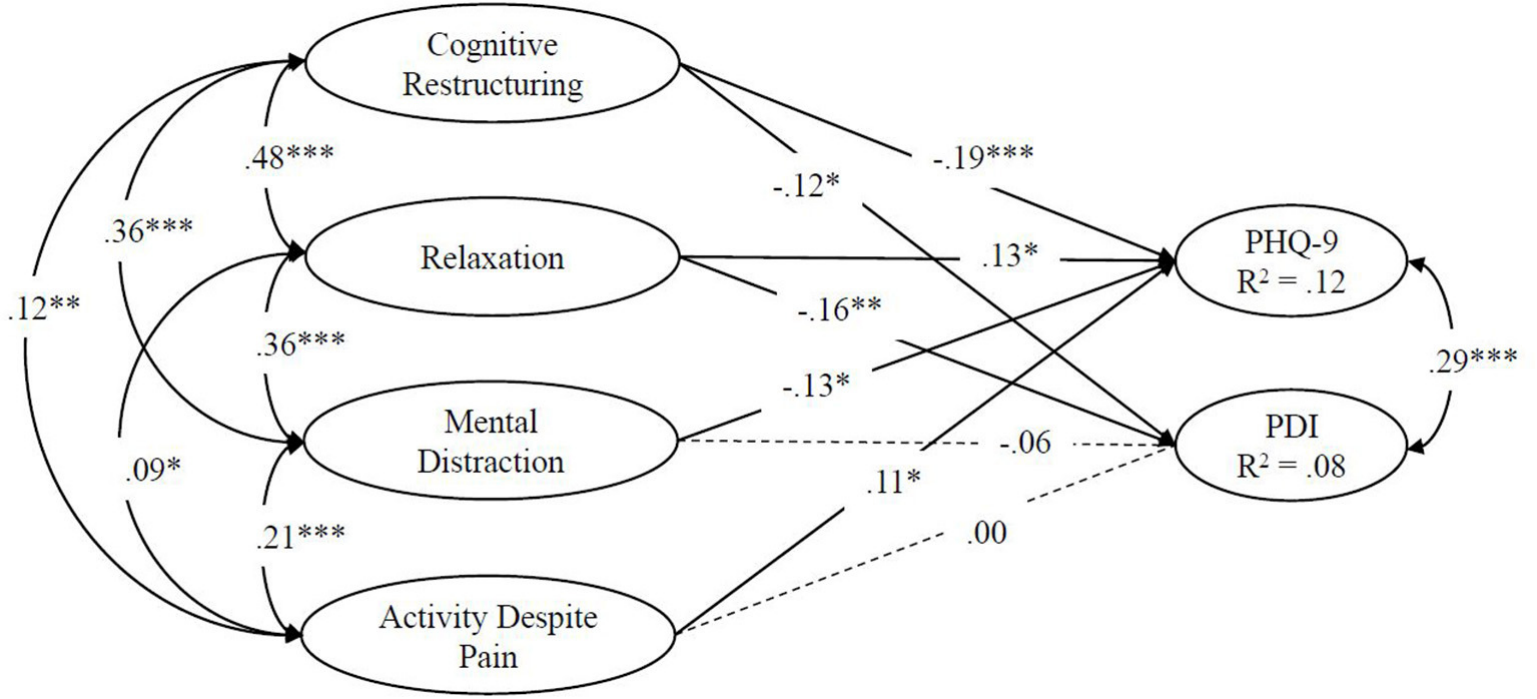
Regressions in a structural equation model on the associations between single indicator latent changes in pain coping skills and changes in disability in Model 1 (clinic A); PDI, Pain Disability Scale; *p < 0.05, **p < 0.001, ***p < 0.00001; estimation with full maximum likelihood; *n* = 754.

Only changes in the use of cognitive restructuring [ß = −0.12, 95% CI = [−0.20, −0.04]] and relaxation technique [ß = −0.16, 95% CI = [−0.25, −0.08]] were independently associated with changes in pain related disability. The independent associations between latent changes in clinic A are depicted in Figure 1.

#### Associations With Changes in Depression

In clinic A, higher reductions in depression were correlated with positive changes in the use of cognitive restructuring [r = −0.28, 95% CI = [−0.35, −0.21]], relaxation technique [r = −0.26, 95% CI = [−0.33, −0.19], and positive changes in the use of mental distraction [r = −0.21, 95% CI = [−0.28; −0.14]] with small to medium effect sizes. Changes in activity despite pain were not correlated with changes in depression [r = 0.05, 95% CI = [−0.03, 0.12]].

Higher reductions in depression were independently associated with positive changes in the use of cognitive restructuring [ß = −0.19, 95% CI = [−0.27, −0.11]], relaxation technique [ß = −0.13, 95% CI = [−0.22, −0.05]], and mental distraction [ß = −0.13, 95% CI = [−0.21, −0.05]] but with negative changes in activity despite pain [ß = 0.11, 95% CI = [0.04, 0.18]].

#### Model Comparisons Between Clinics

In nested model comparisons using only depression as an outcome, constraining regression coefficients to be equal between clinics (Model B) did decrease the performance of the model [X^2^diff(dfdiff = 4, *N* = 1,440) = 10.7, *p* = 0.03]. Further constraining covariances to be equal between clinics (Model C) did not further decrease the performance of the model either [X^2^diff(dfdiff = 45, *N* = 1,440) = 59.4, *p* = 0.19] but constraining all parameter to be equal across clinics did [Model D; X^2^diff(dfdiff = 65, *N* = 1,440) = 118.7, *p* < 0.001]. According to fit indices, the model with equal regression coefficients and covariances between both clinics showed the best fit overall of all constrained models (see Table 3).

**Table 3:** Model fit indices for path models across two clinics.

**Model**	**CFI**	**TLI**	**BIC**	**RMSEA**	**SRMR**
All coefficients free (Null model)	-	-	35434.1	-	-
Regression coefficients equal in both clinics	0.999^†^	0.968	35415.6	0.048	0.005^†^
Regression coefficients and correlations equal	0.997	0.994^†^	35166.2	0.021^†^	0.033
All parameters equal	0.976	0.967	35139.5^†^	0.049	0.055

*Full maximum likelihood estimation of information in structural equation models across two clinics (clinic A with n = 754 and clinic B with n = 686); CFI, comparative fit index; TLI, Tucker–Lewis index; BIC, Bayesian information criterion; SRMR, standardized root mean residual; RMSEA, root mean squared error average;*

† *most favorable value for respective fit index within the block of models for clinic A and B combined; In the combined analyses all parameters were allowed to vary freely by default between the two clinics except for regression coefficients*.

#### Replication of the Associations With Changes in Depression in Clinic B

In clinic B and when data from both clinics were used simultaneously (Model C) most of the effects in clinic A were replicated. However, when only data from clinic B were used, there was a correlation between changes in the use of activity despite pain and changes in depression [r = −0.10, 95% CI = [−0.18, −0.02]] but no direct association [ß = −0.03, 95% CI = [−0.11, 0.05]]. The same was true when data from both clinics were used. Additionally, in clinic B higher reductions in depression were not independently associated with positive changes in the use of mental distraction [ß = −0.09, 95% CI = [−0.18, 0.00]]. All regression coefficients on either change in disability or depression are shown in Table 4.

**Table 4:** Standardized regression weights of pain coping skill changes on temporally concurrent changes in outcomes in clinic A (*n* = 754) clinic B (*n* = 686) and in a model with both datasets combined (*N* = 1,440).

	**Clinic A**		**Clinic B**	**Combined**
**Predictor**	**PDI change**	**PHQ 9 change**	**PHQ 9 change**	**PHQ 9 change**
Cognitive restructuring change	−0.12^*^	−0.19^***^	−0.12^*^	−0.16^***^
Mental distraction change	−0.06	−0.13^*^	−0.09	−0.11^**^
Activity despite pain change	0.00	0.11^*^	−0.03	0.05
Relaxation techniques change	−0.16^**^	−0.13^*^	−0.18^**^	−0.16^***^

* * p < 0.05*,

** * p < 0.001*,

*** *p < 0.00001;*

*Full maximum likelihood estimation of information in structural equation models across two clinics (clinic A with n = 754 and clinic B with n = 686). Changes were modeled as single indicator latent changes*.

#### Sensitivity Analyses

Using multiple imputation instead of full maximum likelihood estimation did not affect whether effects were significant or not. However, using robust maximum likelihood estimation, the independent association of positive changes in the use of mental distraction [ß = −0.09, 95% CI = [−0.18, 0.00]] on pain-related disability in Model A became significant whereas the coefficient did not change.

The pattern of results did not change when age, gender or duration of treatment were introduced into the models as predictors of latent changes.

## Discussion

The first aim of this study was to evaluate the effectiveness of a multidisciplinary pain treatment based on CBT in a large naturalistic sample. Pre–post effect sizes were large to medium and support the effectiveness of inpatient routine care in the treatment of chronic pain. One in three patients reliably improved or recovered from depression and one in five did so for pain-related disability. Second, we investigated the associations of changes in pain coping skills and reductions in pain-related disability and depression. Our data demonstrate small to moderate associations between coping skills for chronic pain and a decrease in both outcomes. Further, we were interested which coping skills in particular are associated with reduced severity of pain-related disability and emotional distress (depression). Cognitive restructuring and relaxation as specific coping skills showed small to moderate associations with improvements in both pain-related disability and depression. The independent association between mental distraction and depression proved to be small but could only be found in one clinic. We found a small negative independent association for the coping skill activity despite pain with respect to depression. However, this could not be replicated in clinic B.

General effect sizes ranged from medium (disability) to large (depression), which is encouraging since current reviews only found small to medium effects for CBT (34). However, the medium to large effect sizes are in line with benchmarks for pre–post effects of randomized CBT trials (77). Since we investigated a multidisciplinary intensive treatment program, slightly higher effect sizes than with CBT alone can be expected (78). In addition, the fact of inpatient treatment may also have contributed to the higher effect sizes, since, for example, patients are not confronted with everyday problems and receive support from fellow patients. In summary the effectiveness in this sample is slightly higher than to be expected from psychological interventions alone, but comparable to similar multidisciplinary interventions and results of randomized CBT trials (78–80). Of note, baseline levels of pain-related disability and inability to work were higher in our study than in these studies. Thus, our data demonstrate promising effects for treating highly disabled pain patients in a multidisciplinary treatment program for chronic pain. However, naturalistic trials often vary in multiple important variables, such as setting, intensity, duration of treatment and symptom severity at baseline. For a more nuanced benchmarking of effect sizes, more data and meta-analytic analyses are needed.

According to our data, both the cognitive approach (cognitive restructuring and mental distraction) and the respondent approach (relaxation) but not the operant approach (activity despite pain) seem to be important for the treatment of chronic pain.

More specific, our results indicate an association between cognitive restructuring and reductions in pain-related disability and depression. This result is in line with a large body of research that highlights the relevance of changing cognitions—especially catastrophizing—in the treatment of chronic pain (81, 82), as they might even have positive effects on pain intensity (83). Even in exposure-based treatments changes in maladaptive thoughts—for example, harm expectations—are of particular importance (84).

Further, the results suggest that changes in relaxation coping skills were associated with changes in disability and depression. Improvements in the use of relaxation were directly associated to a small effect with reductions in pain-related disability and depression. This result supports the fact that relaxation has long been an integral part of the treatment of chronic pain in CBT (85). Furthermore, there is evidence that an increased use of relaxation leads to positive pain-related outcomes (24, 86, 87). Using relaxation as a coping skill may help to reduce pain by reducing muscle tension in affected regions, at least for some forms of pain (88).

The small independent effect of mental distraction on the two outcomes is consistent with our hypothesis and matches with parts of the literature (89), as mental distraction (or attention shifting) is a common treatment component of cognitive therapies (90). However, evidence seems to be heterogeneous (28), with studies supporting possible benefits of distraction from pain (91) and evidence indicating no benefit in a sample similar to the one presented here (30). Given that mental distraction and cognitive restructuring are originally intended to target the same mechanism of change (17), the discrepancy between the associations of these pain coping skills may suggest further differentiation within the cognitive approach.

Contrary to our hypothesis, however, we found no consistent associations between the operant coping skill (e.g., continuing to be active despite pain) and changes in the outcomes. This finding was surprising as operant approaches like graded activity and exposure-based treatments clearly reduce pain-related disability (31, 92) and are associated with a positive mood (93). In addition, positive exposure experiences can be particularly valuable if they lead to cognitive changes, such as may be the case with self-efficacy (94), which is an important protective factor (95). One explanation might be the subscale itself. The items which are intended to measure behavioral coping mainly include a behavioral distraction from pain. It is reasonable to assume that this describes more a reaction to chronic pain like endurance responses including task/pain persistence behavior (96) than actively approaching situations where pain is expected to reduce avoidance behavior like in exposure based treatments (97). Thus, it is possible that the missing association, especially with pain-related disability, is mainly related to the questionnaire's conceptualization of the pain skill activities despite pain.

### Strengths

Main strengths of this study are the large sample size and the use of SEM models for analysis. The results of this study were confirmed by two large, independent and heterogeneous samples of highly disabled chronic pain patients. The use of routine data in a naturalistic setting contributes to a high external validity. The current work identified differences in the associations of changes of different pain coping skills with changes in clinically relevant outcomes. This may provide valuable information on mechanisms of change that are clinically relevant in the treatment of chronic pain. It complements more mechanistic experimental studies by providing information on which pain coping skills might be most relevant for clinical practice. A sufficient discrimination of the pain coping skills and outcome constructs was ensured by asking about pain coping skill use at the level of behavior and by excluding pain coping skill items that implicated positive therapy outcomes. Sensitivity analyses on the sensitive issue of modeling missing data and response distributions were provided.

### Limitations

Because there are only two points of measurement available in the data and the changes happen simultaneously, no conclusions can be drawn on the causal direction of the associations between the changes of pain coping skill use and changes in the outcomes. For example, spontaneous improvements in depressive symptom severity might have induced generally increased levels of activity and therefore increased coping skill use. Implementing more than two points of measurement in routine care would enable researchers to better disentangle the effects and draw stronger causal claims (48) as well as to perform more sophisticated statistical analysis to identify the mechanisms of change (98, 99). In addition, the low and differing reliabilities of the temporal differences in the pain coping skills scales may have reduced the statistical power of the analyses and resulted in a biased pattern of associations between changes. Furthermore, individual therapy was not manualized and treatment plans differed between wards in the weighting of different therapeutic groups apart from the pain-specific group therapy. Patients therefore were likely exposed to different doses of training for the different pain coping skills examined in this work. Unfortunately, information on the medication administered was not available in this data set. The data additionally consist of a mixed pain sample and mechanisms of change might differ between different forms of pain. The emotional and depressive states at the measurement points might not only have influenced depression ratings but also have led to biases in reporting of pain coping skill use. Electronic momentary assessment and electronic diaries might be important tools that could be integrated into routine clinical care to measure pain coping skill use more accurately in future studies (100). Event sampling of coping skill use experiences might also enable researchers to assess the causal effects of different pain coping skills in a naturalistic setting in a similar way to research done on the effect of coping skill use in the context of borderline personality disorder (101).

### Conclusion

We found medium to large effects for a CBT-based multidisciplinary treatment in highly disabled pain patients. The present study supports the importance of coping strategies for reducing pain-related disability and depression. In particular, an increased use of the skills cognitive restructuring, relaxation, and mental distraction appear to be associated with positive treatment outcomes. The focus on the associations of changes in the use of these skills and relevant clinical outcomes in a naturalistic setting complements small-scale experimental studies in identifying the driving mechanisms of change in CBT. Based on our findings, both respondent and cognitive coping skills seem to be relevant mechanisms of change in the treatment of chronic pain. Overall, our findings suggest that not all coping skills might be equally effective. More research is needed to further investigate the important question which skills or mechanisms of change are most effective for pain patients with different sets of characteristics.

## Data Availability Statement

The data analyzed in this study is subject to the following licenses/restrictions: Data sharing is not applicable to this article as only secondary analyses were performed on a routinely collected data set with permission to use for research purposes but without explicit permission of data sharing by the patients. Requests to access these datasets should be directed to Matthias Feldmann, matthias.feldmann@uni-marburg.de.

## Ethics Statement

Ethical review and approval was not required for the study on human participants in accordance with the local legislation and institutional requirements. The patients/participants provided their written informed consent to participate in this study.

## Author Contributions

MF: conceptualization, methodology, statistical analysis, writing—original draft methods and results, review, and editing. HH: conceptualization, methodology, visualization, writing—original draft introduction and discussion, review, and editing. UV: resources, data curation, writing—review and editing, and project administration. RD, TH, and GL: resources, data curation, and writing—review and editing. PH and TK: writing—review and editing. WR: writing—review and editing and supervision. JR: conceptualization, methodology, writing—review and editing, and supervision. E-LB: conceptualization, methodology, writing—review and editing, supervision, and project administration. All authors contributed to the article and approved the submitted version.

## Conflict of Interest

The authors declare that the research was conducted in the absence of any commercial or financial relationships that could be construed as a potential conflict of interest.

## Publisher's Note

All claims expressed in this article are solely those of the authors and do not necessarily represent those of their affiliated organizations, or those of the publisher, the editors and the reviewers. Any product that may be evaluated in this article, or claim that may be made by its manufacturer, is not guaranteed or endorsed by the publisher.
