# Supplementary material for: Cognitive Change and Relaxation as Key Mechanisms of Treatment Outcome in Chronic Pain: Evidence From Routine Care
Source: Front Psychiatry. 2021 Aug 3;12:617871. doi: 10.3389/fpsyt.2021.617871 (PMC8368979; doi:10.3389/fpsyt.2021.617871)
Supplement: Supplementary file 1 [file Table_1.DOCX]

Appendix A

*Bivariate correlations with confidence intervals clinic A.*

| Variable | 1 | 2 | 3 | 4 | 5 | 6 | 7 | 8 | 9 | 10 | 11 |  |  |
| --- | --- | --- | --- | --- | --- | --- | --- | --- | --- | --- | --- | --- | --- |
|  |  |  |  |  |  |  |  |  |  |  |  |  |  |
| 1. Pain related |  |  |  |  |  |  |  |  |  |  |  |  |  |
| disability change |  |  |  |  |  |  |  |  |  |  |  |  |  |
| 2. Depression change | .36** |  |  |  |  |  |  |  |  |  |  |  |  |
|  | [.29, .42] |  |  |  |  |  |  |  |  |  |  |  |  |
| 3. Cognitive | -.22** | -.29** |  |  |  |  |  |  |  |  |  |  |  |
| restructuring change | [-.29, -.15] | [-.36, -.22] |  |  |  |  |  |  |  |  |  |  |  |
| 4. Menatal | -.16** | -.21** | .31** |  |  |  |  |  |  |  |  |  |  |
| distraction change | [-.23, -.09] | [-.28, -.13] | [.25, .38] |  |  |  |  |  |  |  |  |  |  |
| 5. Activitiy despite | -.04 | .05 | .12* | .22** |  |  |  |  |  |  |  |  |  |
| pain change | [-.11, .03] | [-.03, .12] | [.05, .20] | [.15, .29] |  |  |  |  |  |  |  |  |  |
| 6. Relaxation | -.24 | -.26** | .48** | .36** | .09* |  |  |  |  |  |  |  |  |
| techniques change | [-.31, -.17] | [-.33, -.18] | [.42, .54] | [.29, .42] | [.02, .17] |  |  |  |  |  |  |  |  |
| 7. Cognitive | -.04 | .06 | -.61** | -.16** | -.11** | -.21** |  |  |  |  |  |  |  |
| restucturing T1 | [-.11, .04] | [-.02, .13] | [-.65, -.56] | [-.24, -.09] | [-.18, -.04] | [-.28, -.14] |  |  |  |  |  |  |  |
| 8. Mental | .01 | .01 | -.15** | -.42** | -.09* | -.18** | .39** |  |  |  |  |  |  |
| distraction T1 | [-.06, .09] | [-.07, .08] | [-.22, -.08] | [-.48, -.36] | [-.17, -.02] | [-.25, -.11] | [.33, .45] |  |  |  |  |  |  |
| 9. Relaxation | .06 | .04 | -.22** | -.15** | -.09* | -.55** | .45** | .39** |  |  |  |  |  |
| techniques T1 | [-.02, .13] | [-.04, .11] | [-.28, -.14] | [-.22, -.07] | [-.16, -.02] | [-.60, -.49] | [.39, .51] | [.33, .45] |  |  |  |  |  |
| 10. Activity despite | .05 | -.07 | -.07 | -.05 | -.52** | -.02 | .22** | .24** | .13** |  |  |  |  |
| pain T1 | [-.03, .12] | [-.14, .01] | [-.14, .00] | [-.13, .02] | [-.58, -.47] | [-.09, .05] | [.05, .28] | [.18, .31] | [.06, .20] |  |  |  |  |
| 11. Depression T1 | -.04 | -.42** | .08* | .05 | -.06 | .00 | -.29** | -.22** | -.13** | -.07 |  |  |  |
|  | [-.11, .03] | [-.48, -.35] | [.00, .15] | [-.03, .12] | [-.14, .01] | [-.07, .07] | [-.36, -.22] | [-.29, -.14] | [-.20, -.05] | [-.13, .01] |  |  |  |
| 12. Pain related | -.36** | -.10** | .02 | .00 | -.00 | -.01 | -.19** | -.24** | -.11** | -.28** | .50** |  |  |
| disability T1 | [-.42, -.29] | [-.17, -.02] | [-.05, .09] | [-.07, .08] | [-.07, .07] | [-.08, .07] | [-.26, -.12] | [-.31, -.17] | [-.19, -.04] | [-.36, -.22] | [.44, .55] |  |  |
|  |  |  |  |  |  |  |  |  |  |  |  |  |  |

*Note.* Values in square brackets indicate the 95% confidence interval for each correlation. Correlations estimated with full maximum likelihood estimation. Changes modeled as single indicator latent change scores. * indicates *p* < .05. ** indicates *p* < .01.
